# Supplementary material for: Peripheral Immunophenotype in IgG4-Related Disease and Its Association with Clinical Phenotypes and Disease Activity
Source: Cells. 2023 Feb 20;12(4):670. doi: 10.3390/cells12040670 (PMC9954418; doi:10.3390/cells12040670)
Supplement: Supplementary file 1 [file cells-12-00670-s001.zip › cells-2092878-supplementary.pdf]

Supplementary material.

Supplementary Table S1. Antibody information

Antibody	Clone	Catalog no.	
anti-human CD4 PerCP	OKT4	317432	BioLegend
anti-human CD14 APC	M5E2	555397	BD Biosciences
anti-human CD45 RO	UCHL1	304204	BioLegend
anti-human SLAMF7 APC	162.1	331810	BioLegend
anti-human Bcl6 PeCy7	7D1	358512	BioLegend
anti-human CXCR5 APC	J252D4	356908	BioLegend
anti-human CD127 PE	hIL-7R-M21	557938	BioLegend
anti-human ICOS FITC	C398.4A	313506	BioLegend
anti-human CD86 APC	IT2.2	305411	BioLegend
anti-human CD163 PerCP	GHI/61	333626	BioLegend
anti-human CD25 APC	BC96	302610	BD Biosciences
anti-human CD86 FITC	2331	557343	BioLegend
anti-human CD19 APC	HIB19	555415	BD Biosciences
anti-human CD24 FITC	ML5	555427	BD Biosciences
anti-human CD38 PerCP	HB-7	356622	BioLegend
anti-human IL-13 FITC	85BRD	11-713642	In vitrogen
anti-human IL-4 PE	8D4-8	559333	BD Biosciences
anti-human IL-5 PE	JES1-39D10	559332	BD Biosciences
anti-human IL-21 PE	3A3-N2.1	560463	BD Biosciences
anti-human IL-1β PE	JK1B-1	508206	BioLegend
anti-human TGF-β1 ALEXA Fluor 488	259D7C7	560047	BD Biosciences
anti-human IFN-γ PE	4S.B3	559326	BD Biosciences
anti-human Foxp3 PE	259D/C7	560046	BD Biosciences
anti-human Foxp3 ALEXA Fluor 488	PCH101	73-5776-40	e-Bioscience
anti-human IL-9 PE	MH9A3	560807	BD Biosciences
anti-human TNF-α FITC	Mab11	502906	BioLegend
anti-human IL-33 PE	390412	IC3625P	R&D
anti-human IL-10 PE	JES3-19F1	559330	BD Biosciences
anti-human IL-17A PE	SCPL1362	560436	BD Biosciences

Supplementary Table S2. Immunophenotype of the evaluated cell subsets.
**T helper 2 (Th2)**
$CD4^+$/ $CD14^-$/$IL\text{-}4^+$
$CD4^+$/$CD14^-$/$IL\text{-}13^+$
$CD4^+$/$CD14^-$/$IL\text{-}5^+$
**IL-21-producing CD4 T lymphocytes (TCD4 $IL\text{-}21^+$)**
$CD4^+$/ $CD14^-$/$IL\text{-}21^+$
**T helper 9 (Th9)**
$CD4^+$/ $CD14^-$/$IL\text{-}9^+$
**CD4 cytotoxic T lymphocytes ($CD4^+$ CTLs)**
$CD4^+$/ $SLAMF7^+$/$IL\text{-}1\beta^+$/$TGF\text{-}\beta1^+$
**T follicular helper (Tfh)**
Tfh17: $CD4^+$/ $ICOS^+$/$CXCR5^+$/$Bcl6^+$/$IL\text{-}17^+$
Tfh2: $CD4^+$/ $ICOS^+$/$CXCR5^+$/$Bcl6^+$/$IL\text{-}4^+$
Tfh1: $CD4^+$/$ICOS^+$/$CXCR5^+$/$Bcl6^+$/$IFN\text{-}\gamma^+$
T follicular regulatory (Tfr): $CD4^+$/ $ICOS^+$/$CXCR5^+$/$Bcl6^+$/$Foxp3^+$
**Regulatory CD4 T cells**
T regulatory (Tregs): $CD4^+$/$CD25^{hi}$/$CD127^-$/$Foxp3^+$
Type 1 regulatory T (Tr1): $CD4^+$/$CD25^{low}$/$Foxp3^-$/$IL\text{-}10^+$
T helper 3 (Th3): $CD4^+$/$CD25^-$/$Foxp3^-$/$TGF\text{-}\beta1^+$
**IL-10-producing B cells (Bregs)**
$CD19^+$/$CD24^{hi}$/$CD38^{hi}$/$IL\text{-}10^+$
**IL-10-producing regulatory plasmacytoid dendritic cells (pDC $IL\text{-}10^+$)**
$CD86^-$/$CD163^{hi}$/$IL\text{-}10^+$
**M1 monocytes**
$CD86^+$/$CD127^+$/$TNF\text{-}\alpha^+$
**M2 monocytes**
$CD163^+$/$TGF\text{-}\beta1^+$/$IL\text{-}33^+$

Supplementary Table S3. Staining panel						
	**FITC**	**PE**	**Alexa Fluor 488**	**PerCP**	**PECy7**	**APC**
Th2	IL-13	IL-4		CD4		CD14
Th2		IL-5		CD4		CD14
Th9		IL-9		CD4		CD14
IL-21-producing CD4 T cells		IL-21		CD4		CD14
CD4 cytotoxic T lymphocytes		IL-1β	TGF-β1	CD4		SLAMF7
Tfh17	ICOS	IL-17A			Bcl6	CXCR5
Tfh2	ICOS	IL-4			Bcl6	CXCR5
Tfh1	ICOS	IFN-γ			Bcl6	CXCR5
Tf regulatory	ICOS	FoxP3			Bcl6	CXCR5
M1 monocytes	TNF-α	CD127				CD86
M2 monocytes		IL-33	TGF-β1	CD163		CD14
Tregs		CD127	FoxP3	CD4		CD25
Tr1		IL-10	FoxP3	CD4		CD25
Th3		FoxP3	TGF-β1	CD4		CD25
Bregs	CD24	IL-10		CD38		CD19
IL-10-producing regulatory plasmacytoid dendritic cells	CD86	IL-10		CD163		CD14

Supplementary Table S4. Immune cell subsets in proliferative and fibrotic phenotypes

	Proliferative (n = 33)	Fibrotic (n = 10)	p
$CD4^+/CD14^-/IL\text{-}4^+$ (cell/μL)	289.9 (174.6-454.3)	305.1 (106.5-598.5)	0.94
$CD4^+/CD14^-/IL\text{-}13^+$ (cell/μL)	395.2 (257.6-550)	451.7 (178.1-684)	0.83
$CD4^+/CD14^-/IL\text{-}5^+$ (cell/μL)	378.8 (191.1-505)	328 (237.9-448.2)	0.62
$CD4^+/CD14^-/IL\text{-}21^+$ (cell/μL)	387.9 (274.6-695)	359.1 (305.8-402.3)	0.70
$CD4^+/CD14^-/IL\text{-}9^+$ (cell/μL)	417.7 (239.7-646.9)	383.9 (248.9-549.9)	0.45
$CD4^+/SLAMF7^+/IL\text{-}1\beta^+/TGF\text{-}\beta 1^+$ (cell/μL)	1969.8 (1466.8-2354.6)	1556.3 (1136.8-1807.3)	0.26
Tfh ($CD4^+/CXCR5^+/ICOS^+/Bcl6^+$) (cell/μL)	193.3 (113.2-346.8)	170.2 (27.1-204.6)	0.37
Tfh1 (cell/μL)	57.4 (24.7-83)	40.6 (7.5-61.2)	0.23
Tfh2 (cell/μL)	60.9 (28.8-83.1)	39 (7.2-50.3)	0.16
Tfh17 (cell/μL)	29.5 (16.1-79.2)	51.9 (7.6-55.7)	0.81
Tfr (cell/μL)	52.1 (31.4-85.3)	34.7 (6.7-49.3)	0.16
$CD4^+/CD127^-/CD25^{hi}/Foxp3^+$ (Tregs; cell/μL)	392.7 (264.5-481.8)	316.8 (280.9-433.1)	0.50
$CD4^+/CD25^{low}/Foxp3^-/IL\text{-}10^+$ (Tr1; cell/μL)	283.6 (169.3-360.2)	356.9 (264.6-384.8)	0.16
$CD4^+/CD25^-/Foxp3^-/TGF\text{-}\beta 1^+$ (Th3; cell/μL)	280.6 (221.7-371.2)	245.6 (209.1-360.1)	0.64
$CD19^+/CD24^{hi}/CD38^{hi}/IL\text{-}10^+$ (Bregs; cell/μL)	99.5 (62.5-130.3)	81.4 (61.9-118.8)	0.58
$CD86^-/CD163^{hi}/IL\text{-}10^+$ (pDC; cell/μL)	78.1 (58.5-116.5)	79.6 (60.3-114.5)	0.85
$CD163^+/TGF\text{-}\beta 1^+/IL\text{-}33^+$ (M2 Monocytes; cell/μL)	61.3 (53.8-86.1)	66.9 (53.1-86.1)	0.89
$CD86^+/CD127^+/TNF\text{-}\alpha^+$ (M1 Monocytes; cell/μL)	84.3 (55.4-138.6)	106.4 (66.6-169.7)	0.50

Results are presented as medians of cells per microliter for each immune cell subset (interquartile range).

Supplementary Table S5. Immune cell subsets in active vs. inactive IgG4-related disease

	**Active (n = 22)**	**Inactive (n = 21)**	**p**
$CD4^+/CD14^-/IL\text{-}4^+$ (cell/μL)	252.1 (102.9-454.3)	340.4 (217.7-537.2)	0.26
$CD4^+/CD14^-/IL\text{-}13^+$ (cell/μL)	447.4 (244.8-649)	395.1 (257.6-539.6)	0.56
$CD4^+/CD14^-/IL\text{-}5^+$ (cell/μL)	328 (141.4-532)	378.8 (227.5-461.1)	0.86
$CD4^+/CD14^-/IL\text{-}21^+$ (cell/μL)	347.8 (274.6-635.1)	387.9 (330.6-695)	0.61
$CD4^+/CD14^-/IL\text{-}9^+$ (cell/μL)	369.6 (239.7-614,5)	465.3 (358.3-646.9)	0.56
$CD4^+/SLAMF7^+/IL\text{-}1\beta^+/TGF\text{-}\beta1^+$ (cell/μL)	1790.6 (1456-2901.6)	1774.8 (1118.3-2077)	0.25
Tfh ($CD4^+/CXCR5^+/ICOS^+/Bcl6^+$) (cell/μL)	274.4 (146.3-370.8)	169.6 (112.9-209.9)	0.18
Tfh1 (cell/μL)	59.9 (23.6-84.9)	51.1 (19.6-61.6)	0.46
Tfh2 (cell/μL)	65.5 (35.1-113.2)	39.2 (24.9-68.1)	0.13
Tfh17 (cell/μL)	58.2 (21.8-119.9)	24.8 (14.3-44.2)	0.03
Tfr (cell/μL)	68.6 (29.7-101.5)	45.4 (30.1-55.9)	0.30
$CD4^+/CD127^-/CD25^{hi}/Foxp3^+$ (Tregs; cell/μL)	373.6 (262.6-488.8)	333.8 (299.2-448.5)	0.66
$CD4^+/CD25^{low}/Foxp3^-/IL\text{-}10^+$ (Tr1; cell/μL)	363.5 (292.2-488.8)	227.3 (153.6-283.6)	<0.001
$CD4^+/CD25^-/Foxp3^-/TGF\text{-}\beta1^+$ (Th3; cell/μL)	297.8 (242.6-454.1)	244.8 (181.9-303.8)	0.02
$CD19^+/CD24^{hi}/CD38^{hi}/IL\text{-}10^+$ (Bregs; cell/μL)	92.3 (61.6-131)	95.2 (64.7-118.8)	0.62
$CD86^-/CD163^{hi}/IL\text{-}10^+$ (pDC; cell/μL)	107.1 (74.2-128.6)	62.2 (53.5-81.8)	0.009
$CD163^+/TGF\text{-}\beta1^+/IL\text{-}33^+$ (M2 Monocytes; cell/μL)	76.9 (58.5-102.1)	58.7 (49-77.5)	0.04
$CD86^+/CD127^+/TNF\text{-}\alpha^+$ (M1 Monocytes; cell/μL)	116.8 (74.7-155.5)	77.2 (52.2-99.8)	0.02

Results are presented as medians of cells per microliter for each immune cell subset (interquartile range).

Supplementary Table S6. Immune cell subsets in atopic vs. non-atopic IgG4-related disease			
	**Atopic (*n* = 11)**	**Non-atopic (*n* = 32)**	***p***
$CD4^+/CD14^-/IL\text{-}4^+$ (cell/μL)	453.3 (276.8-774.5)	273.6 (108.7-352.4)	0.06
$CD4^+/CD14^-/IL\text{-}13^+$ (cell/μL)	480.4 (262.9-731.4)	388.8 (251.2-579.5)	0.50
$CD4^+/CD14^-/IL\text{-}5^+$ (cell/μL)	502.3 (296.5-630.4)	297.8 (188.7-451.8)	0.08
$CD4^+/CD14^-/IL\text{-}21^+$ (cell/μL)	549.9 (224.8-897.4)	372.9 (296.4-554.4)	0.61
$CD4^+/CD14^-/IL\text{-}9^+$ (cell/μL)	546.1 (259.7-729)	397.7 (244.3-569.3)	0.50
$CD4^+/SLAMF7^+/IL\text{-}1\beta^+/TGF\text{-}\beta1^+$ (cell/μL)	1771 (1610.3-2287)	1791 (1180.4-2307)	0.46
Tfh ($CD4^+/CXCR5^+/ICOS^+/Bcl6^+$) (cell/μL)	126.5 (57.3-183.8)	207.3 (129.7-344)	0.11
Tfh1 (cell/μL)	44.7 (11.7-58)	59.7 (29.3-84)	0.12
Tfh2 (cell/μL)	35.2 (20.1-44.8)	62.2 (32.3-82.9)	0.12
Tfh17 (cell/μL)	21.2 (7.7-40.2)	47.9 (20.7-79.9)	0.14
Tfr (cell/μL)	32.1 (14.6-44.3)	54 (32.3-93.4)	0.04
$CD4^+/CD127^-/CD25^{hi}/Foxp3^+$ (Tregs; cell/μL)	392.7 (291.9-492.5)	342.8 (257-471.4)	0.63
$CD4^+/CD25^{low}/Foxp3^-/IL\text{-}10^+$ (Tr1; cell/μL)	359.6 (260.7-427.8)	272.7 (171.3-371.2)	0.24
$CD4^+/CD25^-/Foxp3^-/TGF\text{-}\beta1^+$ (Th3; cell/μL)	318.2 (241.2-392.3)	250.9 (219.5-347.2)	0.48
$CD19^+/CD24^{hi}/CD38^{hi}/IL\text{-}10^+$ (Bregs; cell/μL)	117.3 (75-127.5)	90 (61.7-127.6)	0.40
$CD86^-/CD163^{hi}/IL\text{-}10^+$ (pDC; cell/μL)	81.8 (77.2-118.8)	75.5 (54.5-114.4)	0.38
$CD163^+/TGF\text{-}\beta1^+/IL\text{-}33^+$ (M2 Monocytes; cell/μL)	85.8 (68.3-97.8)	60 (52.8-89.7)	0.08
$CD86^+/CD127^+/TNF\text{-}\alpha^+$ (M1 Monocytes; cell/μL)	99.8 (69.2-136.3)	86.9 (57.2-151)	1.00

Results are presented as medians of cells per microliter for each immune cell subset (interquartile range).

Supplementary Table S7. Immune cell subsets in treated vs. untreated IgG4-related disease			
	**Treated** (***n*** **= 27)**	**Untreated** (***n*** **= 16)**	**p**
$CD4^+/CD14^-/IL\text{-}4^+$ (cell/μL)	308.3 (186.3-592.2)	255.7 (96.4-416.7)	0.30
$CD4^+/CD14^-/IL\text{-}13^+$ (cell/μL)	414.4 (271.6-741)	311.6 (211.4-519.5)	0.24
$CD4^+/CD14^-/IL\text{-}5^+$ (cell/μL)	363.7 (209.3-513.7)	306.4 (183.3-480.2)	0.86
$CD4^+/CD14^-/IL\text{-}21^+$ (cell/μL)	426.1 (301.2-739.7)	334.6 (257.4-395.1)	0.16
$CD4^+/CD14^-/IL\text{-}9^+$ (cell/μL)	465.3 (244.3-660.8)	389.9 (250.7-471.4)	0.54
$CD4^+/SLAMF7^+/IL\text{-}1\beta^+/TGF\text{-}\beta1^+$ (cell/μL)	2050 (1127.5-2718.6)	1720.1 (1453.2-1983.2)	0.42
Tfh ($CD4^+/CXCR5^+/ICOS^+/Bcl6^+$) (cell/μL)	174.1 (77.7-325.5)	188.6 (149.3-344)	0.51
Tfh1 (cell/μL)	53.6 (18.4-76.5)	55.4 (39.5-75.1)	0.69
Tfh2 (cell/μL)	48.4 (23.8-72.9)	45.2 (35.7-83.4)	0.53
Tfh17 (cell/μL)	25.8 (11.5-68.2)	52.5 (26.6-77.8)	0.23
Tfr (cell/μL)	45.4 (22.3-76.9)	50.7 (32.4-85.1)	0.51
$CD4^+/CD127^-/CD25^{hi}/Foxp3^+$ (Tregs; cell/μL)	392.7 (293-481.3)	303.5 (267.2-461.5)	0.34
$CD4^+/CD25^{low}/Foxp3^-/IL\text{-}10^+$ (Tr1; cell/μL)	351 (219-417.3)	246.6 (189-313.5)	0.11
$CD4^+/CD25^-/Foxp3^-/TGF\text{-}\beta1^+$ (Th3; cell/μL)	276.5 (229.8-415.5)	266.6 (219.5-321.8)	0.56
$CD19^+/CD24^{hi}/CD38^{hi}/IL\text{-}10^+$ (Bregs; cell/μL)	95.2 (63.4-134.4)	93.1 (62-121.5)	0.70
$CD86^-/CD163^{hi}/IL\text{-}10^+$ (pDC; cell/μL)	78 (55.9-115.5)	79.6 (60.8-117.8)	0.82
$CD163^+/TGF\text{-}\beta1^+/IL\text{-}33^+$ (M2 Monocytes; cell/μL)	77.5 (52.4-101.6)	59.4 (55.8-66.7)	0.16
$CD86^+/CD127^+/TNF\text{-}\alpha^+$ (M1 Monocytes; cell/μL)	91.5 (54.7-129.4)	88.8 (65.4-149.6)	0.66

Results are presented as medians of cells per microliter for each immune cell subset (interquartile range).

Supplementary Table S8. Immune cell subsets in non-treated IgG4-related disease and healthy subjects

	Non-treated (n = 16)	Controls (n = 12)	p
$CD4^+/CD14^-/IL\text{-}4^+$ (cell/µL)	255.7 (96.3-416.6)	89.2 (66.3-111.9)	0.002
$CD4^+/CD14^-/IL\text{-}13^+$ (cell/µL)	311.6 (211.4-519.5)	89.4 (72.7-152.4)	0.001
$CD4^+/CD14^-/IL\text{-}5^+$ (cell/µL)	306.4 (183.3-480.2)	89.6 (65.3-105.4)	<0.001
$CD4^+/CD14^-/IL\text{-}21^+$ (cell/µL)	334.6 (257.3-395)	131.8 (95.4-153)	<0.001
$CD4^+/CD14^-/IL\text{-}9^+$ (cell/µL)	389.9 (250.7-471.4)	94 (64-217)	<0.001
$CD4^+/SLAMF7^+/IL\text{-}1\beta^+/TGF\text{-}\beta1^+$ (cell/µL)	1720 (1453.2-1983.2)	454.7 (398.6-630.8)	<0.001
Tfh ($CD4^+/CXCR5^+/ICOS^+/Bcl6^+$) (cell/µL)	188.7 (149.3-344)	20.6 (19.3-21.7)	<0.001
Tfh1 (cell/µL)	55.4 (39.5-76.2)	5.6 (4.5-6.1)	<0.001
Tfh2 (cell/µL)	45.2 (35.7-83.3)	4.8 (4.5-5.4)	<0.001
Tfh17 (cell/µL)	52.5 (26.6-77.8)	4.6 (3.9-6)	<0.001
Tfr (cell/µL)	50.7 (32.4-85.1)	5.5 (5.3-6.2)	<0.001
$CD4^+/CD127^-/CD25^{hi}/Foxp3^+$ (Tregs; cell/µL)	303.5 (267.2-461.5)	244.1 (158.7-291.5)	0.01
$CD4^+/CD25^{low}/Foxp3^-/IL\text{-}10^+$ (Tr1; cell/µL)	246.5 (189-313.5)	140.8 (80.4-192.2)	0.003
$CD4^+/CD25^-/Foxp3^-/TGF\text{-}\beta1^+$ (Th3; cell/µL)	266.6 (219.5-321.8)	98 (70.6-137)	<0.001
$CD19^+/CD24^{hi}/CD38^{hi}/IL\text{-}10^+$ (Bregs; cell/µL)	93.2 (62-121.5)	56.1 (44.4-70.9)	0.004
$CD86^-/CD163^{hi}/IL\text{-}10^+$ (pDC; cell/µL)	79.6 (60.8-117.8)	63.1 (49.8-77.1)	0.04
$CD163^+/TGF\text{-}\beta1^+/IL\text{-}33^+$ (M2 Monocytes; cell/µL)	59.4 (55.8-66.7)	37.9 (29.8-49.3)	<0.001
$CD86^+/CD127^+/TNF\text{-}\alpha^+$ (M1 Monocytes; cell/µL)	88.8 (65.4-149.7)	43.7 (39.8-49.5)	<0.001

Results are presented as medians of cells per microliter for each immune cell subset (interquartile range).

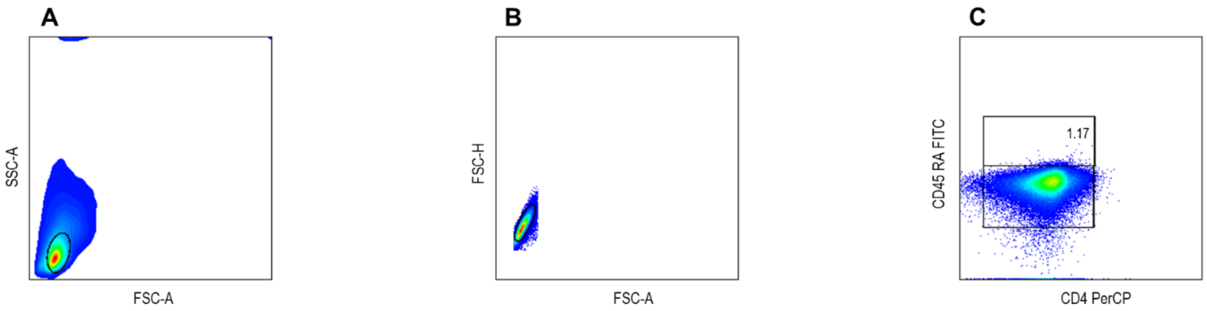

Supplementary Figure S1. Representative dot plot of the CD4+T memory cell isolation.

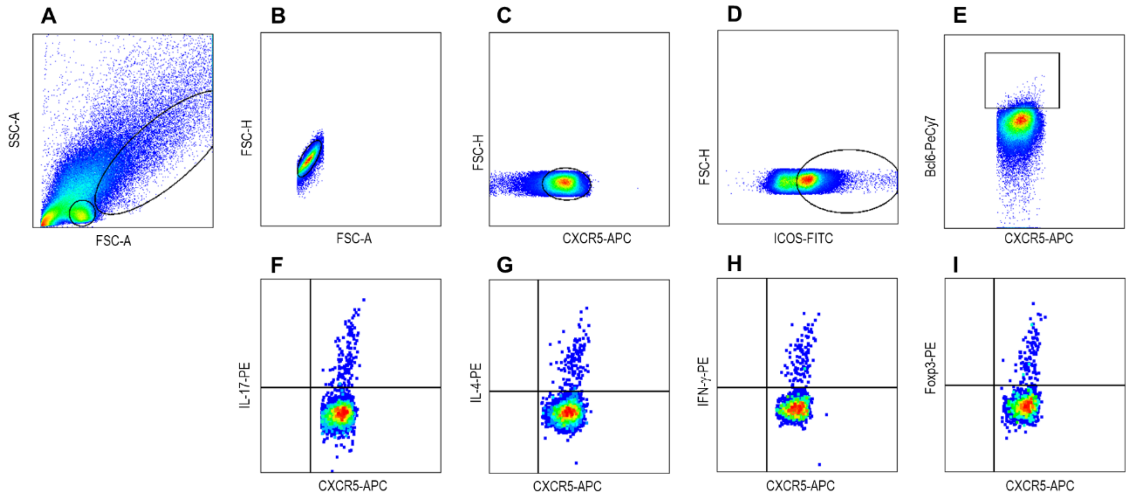

Supplementary Figure S2. Percentage of circulating Tfh cells in a patient with IgG4-related disease. (A) Representative plot from the lymphocyte subpopulation was gated. (B) An electronic gate was made for singlets. (C) From the latter an electronic gate was made for CXCR5+ cells. (D) From the gate CXCR5+/ICOS+ cells were determined. (E) From the gate CXCR5+/ICOS+/Bcl6+ cells were evaluated, and an electronic gate was made for triple-positive cells. (F) From the gate CXCR5+/ICOS+/Bcl6+/IL-17+ cells were determined. (G) From the gate CXCR5+/ICOS+/Bcl6+/IL-4+ cells were determined. (H) From the gate CXCR5+/ICOS+/Bcl6+/IFN-g+ cells were determined. (I) From the gate CXCR5+/ICOS+/Bcl6+/Foxp3+ cells were selected.

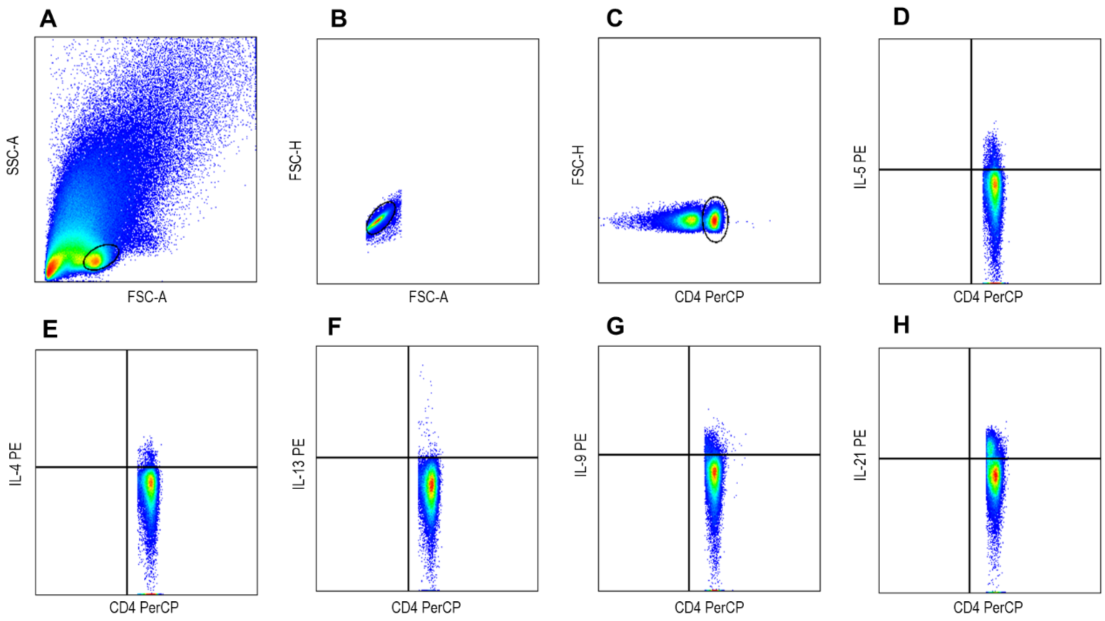

Supplementary Figure S3. Percentage of circulating CD4 effector T cells in a patient with IgG4-related disease. (A) Representative plot from the lymphocyte subpopulation was gated. (B) An electronic gate was made for singlets. (C) From the latter, an electronic gate was made for CD4+ cells. (D) From the gate of figure B, IL-5+ cells were determined. (E) From the gate of figure B, IL-4+ cells were evaluated. (F) From the gate of figure B, IL-13+ cells were determined. (G) From the gate of figure B, IL-9+ cells were determined. (H) From the gate of figure B, IL-21+ cells were determined.

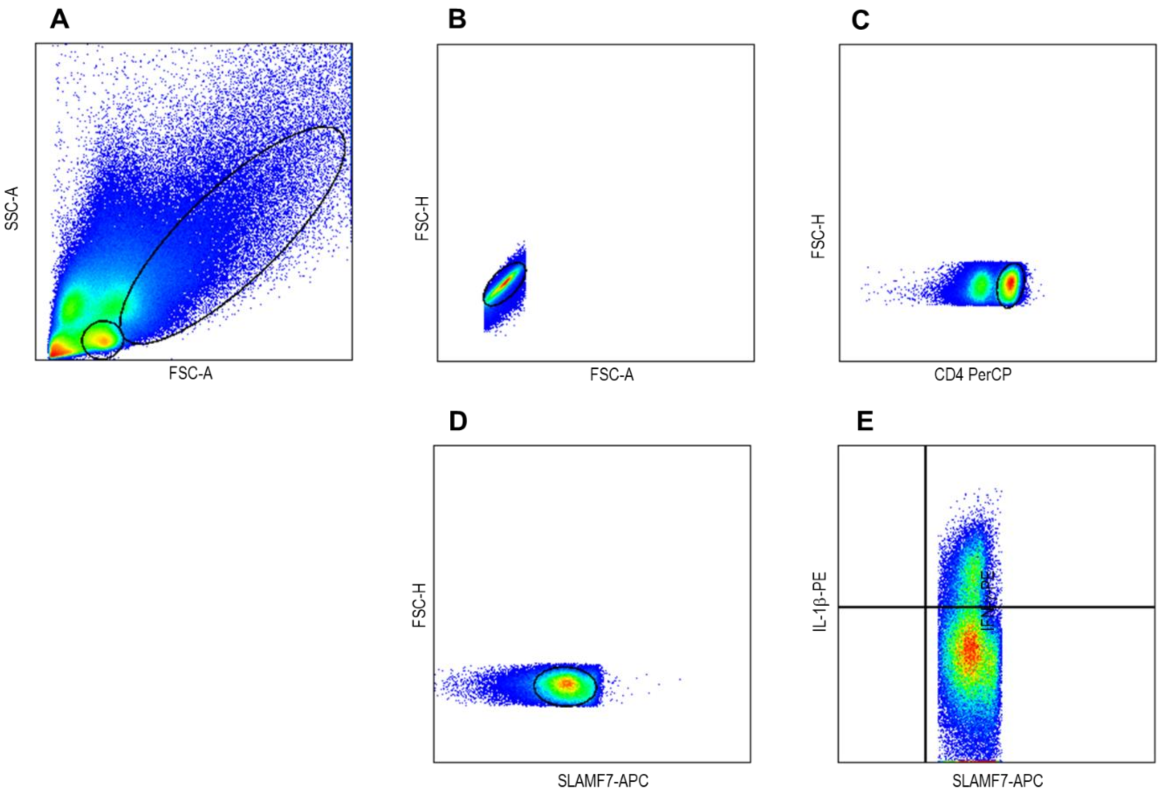

Supplementary Figure S4. Percentage of circulating CD4+ cytotoxic T lymphocytes in a patient with IgG4-related disease. (A) Representative plot from the lymphocyte subpopulation was gated. (B) An electronic gate was made for singlets. (C) From the latter an electronic gate was made for CD4+ cells. (D) From the gate of CD4+ cells/SLAMF7+ cells were determined. (E) From the gate of figure D, IL-β+/TGF-β1+ cells were evaluated.

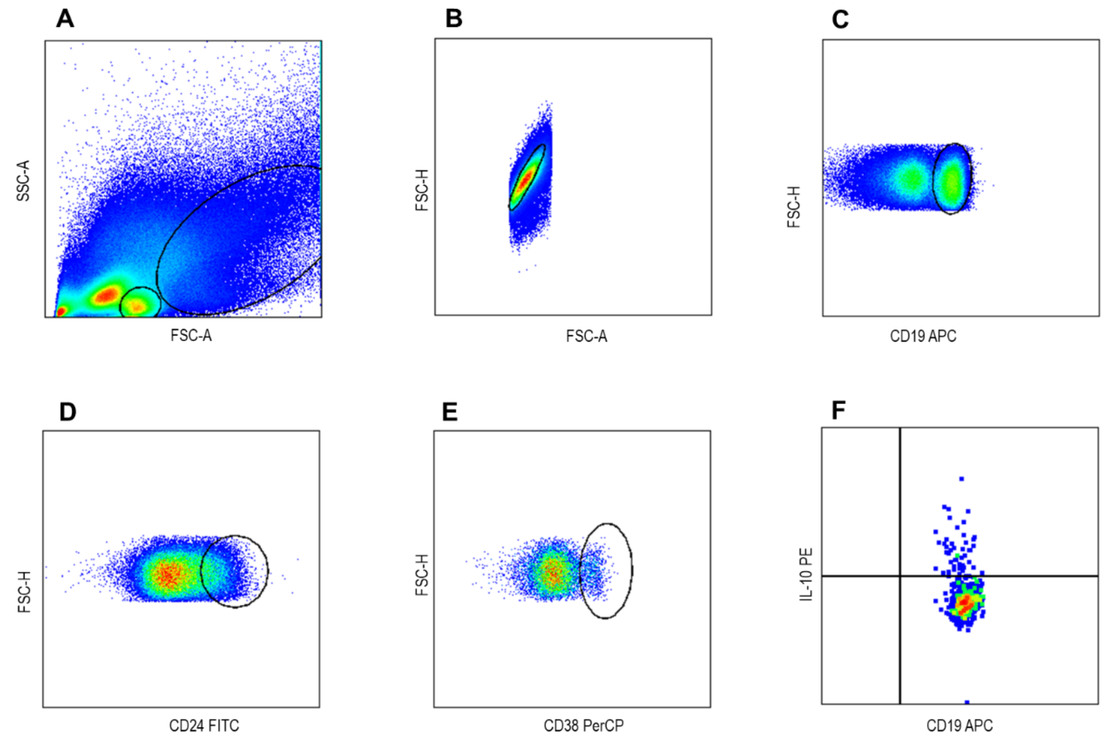

Supplementary Figure S5. Percentage of circulating IL-10-producing B cells in a patient with IgG4-related disease. (A) Representative plot from the lymphocyte subpopulation was gated. (B) An electronic gate was made for singlets. (C) From the latter, an electronic gate was made for CD19+ cells. (D) From the gate of CD19+ cells, CD24+ cells were determined. (E) From the gate of CD19+/CD24+ cells, CD38+ cells were evaluated. (F) From the gate of CD19+/CD24+/CD38+/IL-10+, cells were evaluated.
